# Presenting Symptoms and Disease Severity in Multiple Sclerosis Patients

**DOI:** 10.3390/neurolint13010002

**Published:** 2021-01-08

**Authors:** Jason Ledesma, Padma Priya Puttagunta, Shayan Torabi, Kristen Berube, Eric Tamrazian, Diamond Garcia, Bijal Kirit Mehta

**Affiliations:** 1 Department of Neurology, The Lundquist Institute, Torrance, CA 90504, USA; puttagunta@labiomed.org (P.P.P.); storabi@labiomed.org (S.T.); kberube@labiomed.org (K.B.); tamrazianeric@gmail.com (E.T.); dgarcia@lundquist.org (D.G.); bijal@yahoo.com (B.K.M.); 2 Los Angeles County Harbor-UCLA Medical Center, Department of Neurology, David Geffen School of Medicine at UCLA, Torrance, CA 90509, USA

**Keywords:** multiple sclerosis (MS), presenting symptoms, disease severity, lesion volumes, expanded disability status scale (EDSS), balance, vision, sensory function, motor function

## Abstract

Introduction: The study aims to determine an association between presenting symptoms in multiple sclerosis and measures of disease severity, including the expanded disability status score (EDSS) and MRI based lesion volumes. Methods: Data was collected as part of a larger 3 year MS study, from 2014 to 2017, to compare Vitamin A levels and MS progression. All data was collected from a single clinical site. Demographic data as well as date of diagnosis and use of disease modifying therapies. Patients not able to obtain MRIs or lab tests and histories of vitamin abnormalities were excluded from the study. 29 patients met inclusion criteria. We chose presenting symptoms of vision, balance, sensory function, and motor function as these represented the most common manifestations of the disease and mirror the domains of the EDSS, which is the most commonly used scale for MS disease severity. We also included neuroimaging based lesion volume as another objective measure for comparison. Results: Although duration of disease was different between comparator groups, no significant difference was found between them when EDSS and lesion volumes were compared. There was a difference in lesion volumes when comparing those patients that had presenting symptoms of visual changes or balance symptoms with those presenting with sensory changes. Conclusions: This study supports the notion that presenting symptoms are not associated with EDSS independent disease duration. It also verifies that severity of disease is not associated with lesion volumes. However, sensory symptoms as a presenting symptom was associated with less lesion volumes in our study.

## 1. Introduction

Multiple Sclerosis (MS) is an autoimmune demyelinating disease of the central nervous system (CNS). Diagnosing this idiopathic, inflammatory disease of the CNS is based on the presence of lesions in the brain and spine, often under the diagnostic mantra of being “separated in space and time”. These lesions are believed to be the result of autoreactive B cell or T cells which “conduct” the destruction of myelin, oligodendrocytes, and neurons [1,2]. However, the initiating etiology of the disease is unknown. As a result, patients have asymmetric responses to the different treatment modalities and all indication of resulting periods of cumulative remission or relapse often do not provide definitive quantitative evidence [3]. To estimate a patient’s degree of disease severity, the Expanded Disability Status Score (EDSS) is often used. The EDSS assessment disregards patients’ reported symptoms and instead focuses on the results of neurological examination of “Function Systems” [4,5]. Additionally, many recent studies have implemented the calculation of lesion volumes via MRI scan analysis to supplement understanding of a patient’s disease severity [4,6,7].

Cumulative EDSS assessment and quantitative lesion analysis have helped to clarify that the disease progression of MS is unique to each patient. Presenting symptoms can take the form of optic neuritis, degradation of cerebellar and pyramidal function, and myelopathy, as examples [3,8,9,10,11,12]. The current study aims to establish an association between early presenting symptoms relating to vision, motor function, sensory function, and balance and the measures of disease severity mentioned above (EDSS scores, lesion volume) in relapse-remitting MS (RRMS) patients.

## 2. Materials and Methods

The Internal Review Board approval, including ethics committee review was on 5/13/2014 (project #30174-01). Patients were recruited and consented as part of a 3-year study concerning the relationship between vitamin A level and MS progression, with a majority of patients enrolled through a single MS clinic at a large county safety net hospital. All included patients were diagnosed with relapse-remitting MS, of varying severity (EDSS MIN: 1.5, EDSS MAX: 7.5, EDSS MEAN: 4). Patients’ vitamin D levels were on average maintained at normal levels (≥30 ng/mL), with supplements being prescribed when low levels were detected (<20 ng/mL) as part of the standard of care. The patients’ demographic and medical history were collected prior to enrollment, including estimated date of diagnosis and type of MS medication. For this study, duration of disease was considered the time between date of diagnosis and start of the study, or baseline. Although patients may have been enrolled prior to official diagnosis, patients taking or started on copaxone, an interferon-beta disease modifying therapy, or an oral MS therapy treatment (Aubagio, Tecfidera, Fingoliomod) were selected. Exclusion criteria also included inability to have MRI studies or lab draws, and prior history of disorders associated with abnormal vitamin D or vitamin A levels. This included patients with abnormally low vitamin A levels (<30 micrograms/dL).

**Figure 1:**
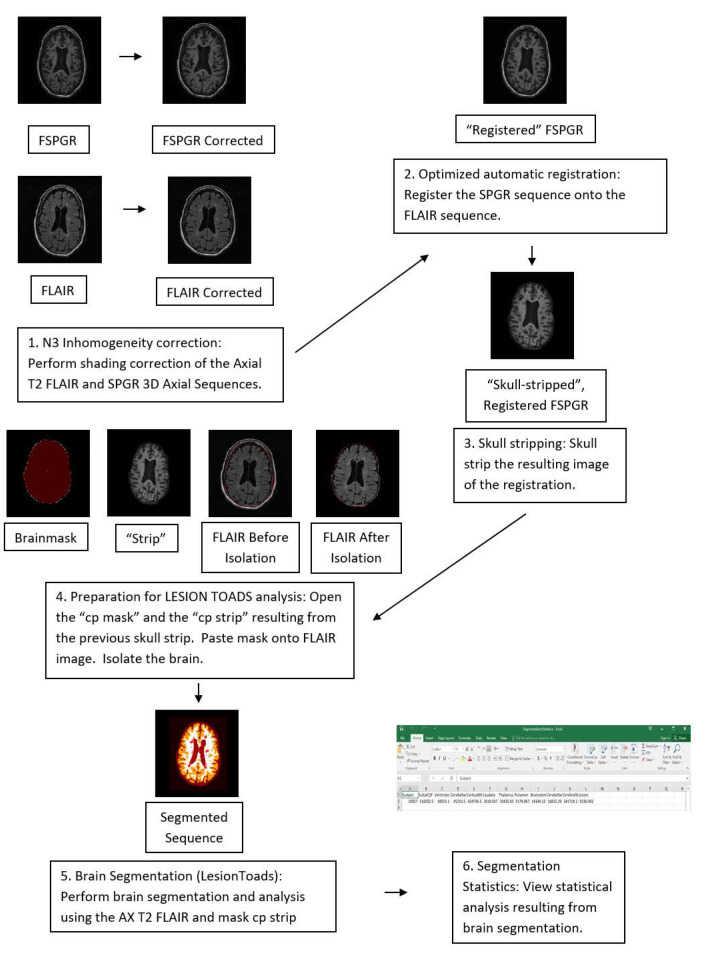
Software based Lesion Analysis “Work Flow”.

The consenting process and baseline assessment occurred between 2014 and 2016. Patients were reviewed by a neurologist and given a baseline EDSS score during their first visit. All patients underwent an initial baseline MRI scan of the brain and cervical spine via a 3 Tesla MRI. Twenty-nine patients met the inclusion criteria. All selected patients had MRI scans with little to no artifact noted, a disease duration between 1 and 15 years, and presenting symptoms involving impairment of vision, balance, sensory function, or motor function. These symptoms were chosen because they were most prevalent in the patient population, and because they are representative of primary common manifestations of the disease, particularly at onset [3,11]. The Java application Medical Imaging Processing, Analysis, and Visualization (MIPAV, Version 7.40, Center of Information Technology and National Institute of Health, Bethesda, MD) was used to calculate lesion volume. MIPAV’s “LesionToads” plugin (TOADS-CRUISE 2014 May 05 R4c release) is capable of performing a brain segmentation (mm^3^). FSPGR 3D Axial and T2 FLAIR Axial series were used in this analysis, with the FSPGR imaging serving as the T1 series to be referenced by the LesionToads software (TOADS-CRUISE 2014 May 05 R4c release). Images in these series were corrected for shading, co-registered, and skull-stripped in MIPAV before being assessed via the LesionToads function of the TOADS-CRUISE plugin software. See Figure 1 [13,14,15].

During statistical analysis, patients were divided by their presenting symptoms into four groups: visual (*n* = 11), balance (*n* = 6), sensory (*n* = 8), and motor (*n* = 4). See Table 1. Patients were also divided by disease duration and medication type to account for the effects of these variables. Average years of disease duration were compared with *t*-tests and Mann–Whitney U tests between patients with disease duration greater than 5 years (*n* = 13) and patients with disease duration less than 5 years (*n* = 16). Average EDSS scores and average lesion volumes were also compared across these two groups. To account for disease modifying therapy, patients were divided into the interferon group (*n* = 15), the tecfidera group (*n* = 4), and the copaxone group (*n* = 10). *t*-tests and Mann–Whitney tests were similarly used to assess differences in EDSS score and lesion volume, and the addition of an ANOVA test was incorporated to evaluate measures of disease severity across all groups. Once the effects of confounding variables were determined, EDSS scores and lesion volumes were compared across the visual, balance, sensory, and motor groups with *t*-tests, Mann–Whitney tests, and ANOVA tests. In all cases, *p* values less than 0.05 were considered significant.

## 3. Results

**Figure 2:**
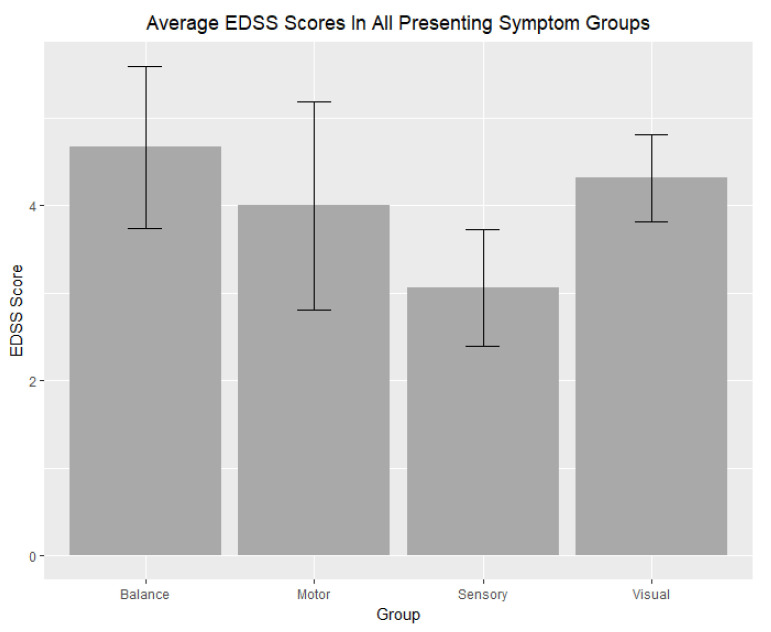
Average EDSS Scores In All Presenting Symptom Groups. The average EDSS scores in the Balance (4.66667), Visual (4.31818), Motor (4.0000), and Sensory (3.06250) groups were not significant different. Error bars represent the standard error of the mean in the Balance (0.92761), Visual (0.49668), Motor (1.19024), and Sensory (0.67107) groups.

**Figure 3:**
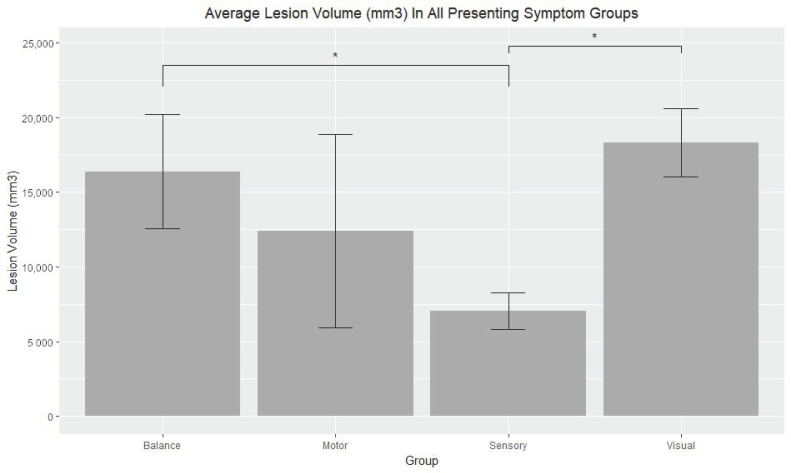
Average Lesion Volume (mm^3^) In All Presenting Symptom Groups. The average lesion volumes in the Balance (16,377.06 mm^3^) and Visual (18,297.72 mm^3^) groups were significantly different than the average lesion volume of the Sensory (7034.21 mm^3^) group (significant differences noted by asterisks). No significant differences were determined when comparing average lesion volume of the Motor (12,383.10 mm^3^) group with the other three presenting symptom groups. *Error bars* represent the standard error of the mean in the Balance (3821.22 mm^3^), Visual (2290.53 mm^3^), Motor (6468.12 mm^3^), and Sensory (1208.95 mm^3^) groups.

Patients with disease duration greater than 5 years had significantly greater average years of disease than the patients with disease duration less than 5 years (*t*-test: *p* < 0.001, U-test: *p* < 0.0001). EDSS scores (*t*-test: *p* = 0.85, U-test: *p* = 0.67) and lesion volume (*t*-test: *p* = 0.42, U-test: *p* = 0.6455), however, were not significantly different. With regards to the medication groups, no significant differences were detected with respect to EDSS scores and lesion volumes (all *p* > 0.05). The average EDSS scores in the Balance (4.66667), Visual (4.31818), Motor (4.0000), and Sensory (3.06250) groups were not significant different (*p* = 0.43). See Figure 2. Significant differences in lesion volume were seen when comparing the visual and sensory groups (*t*-test: 0.001, U-test: 0.001), and when comparing the balance and sensory groups (*t*-test: 0.02, U-test: *p* > 0.05). See Figure 3. An ANOVA test across all presenting symptom groups also showed significance (*p* = 0.03). In all other comparisons concerning presenting symptom groups, no significant differences were found (all *p* > 0.05). See Table 2.

## 4. Discussion

The significant difference in average years between the greater than 5 year group and the less than 5 year group coupled with the lack of significance in EDSS score and lesion volume seen when comparing these same groups proposes that disease duration in our comparison groups did not influence disease severity. Patients with significantly longer duration of disease did not also have significantly greater EDSS scores or lesion volumes. Similarly, significant differences were not found when comparing the different medication type groups, indicating that medication had no influence on EDSS score and lesion volume. In general, significant differences were not seen when comparing presenting symptom groups, illustrating that there is little correlation between presenting symptom type and disease severity (*p* > 0.05). Exceptions were seen when comparing lesion volume in the visual group and the sensory group and when comparing lesion volume in the balance group and the sensory group. The sensory group had lower lesion volume level in both comparisons with an average of 7034 mm^3^. The visual and balance groups had average lesion volumes of 18,297 mm^3^ and 16,377 mm^3^, respectively. This suggests that lesion size may influence different symptoms of MS disproportionately, and that overall degrees of neurological disability does not have a proportional relationship to lesion size. Specifically, impairment in sensory and motor functionality may be more affected by lesion volume than visual and balance functionality. Furthermore, EDSS scores, and therefore overall neurological disability, were not significantly different across groups, further suggesting that the effects of lesion size may differ depending on the individual’s disease course.

It may be possible to create a scale for lesion volume scores based upon the principle that lesion volume and location may be proportional to different modes of CNS damage. Thus, a series of ratios could be created to be represented by a proposed lesion volume “score”. For example, a smaller lesion volume causing significant sensory impairment could be equivalent in severity to a larger lesion volume causing significant visual impairment. Therefore, a set of lesion volume-to-degree symptom severity ratios could be devised and implemented to help to better characterize quantitatively a patient’s MS severity.

The study’s lack of significant findings can be attributed to the nature and number of the patients, as only 29 patients were included for analysis. The power of the study’s findings is minimal and at best preliminary. Though the confounding variable of disease duration was accounted for, the EDSS score and lesion volume calculated for each patient occurred at the baseline of the study, not at the time during which presenting symptoms initially occurred.

## 5. Conclusions

Overall, the study did not find a significant association between type of presenting symptom and disease severity. The sensory group, on average, had a significantly lesser average lesion volume than the visual and balance groups. This suggests that sensory deficits may be more susceptible to MS lesions, as greater lesion volumes may be necessary to result in visual or balance-related deficits. In general, however, a patient presenting symptom did not correlate to EDSS score or lesion volume in any significant way, as comparisons between the visual, balance, sensory, and motor groups illustrated. Failure to determine more conclusive findings may in part be attributed to the inability of EDSS scores to serve as proper representatives of a patient’s disease condition, as EDSS scores for each patient may vary according to a neurologist’s subjectivity and acumen. To further characterize the relationship between presenting symptoms and disease severity, further analysis is required with larger populations.

## Figures and Tables

**Table 1:** Patient Demographics: The study’s patient population consisted of males and females, Caucasians, Hispanic Americans, African Americans, and Asian Americans. The patients’ average age was approximately 40 years old. Patients of different demographic backgrounds were not equally distributed across presenting symptom groups.

	All Patients	Visual Group	Balance Group	Sensory Group	Motor Group
Number of Males	12	6	1	4	1
Number of Females	17	5	5	4	3
Number of Caucasians	5	2	0	2	1
Number of Hispanic Americans	12	5	2	3	2
Number of African Americans	9	3	4	1	1
Number of Asian Americans	3	1	0	2	0
Average Age	40.586	38.364	46.167	34.625	50.25

**Table 2:** Overview of comparisons between presenting symptom groups. *t*-tests, U-tests, and ANOVA tests were performed to compare EDSS scores and lesion volumes across presenting symptom groups. Significant differences were seen when comparing lesion volume in the Visual and Sensory groups, lesion volume in the Balance and Sensory groups, and lesion volume in all groups (*p* < 0.05).

Groups Assessed	T Test (EDSS)	U Test (EDSS)	ANOVA (EDSS)	T Test (LV)	U Test (LV)	ANOVA (LV)
Visual vs. Balance	0.72	0.80	NA	0.65	0.65	NA
Visual vs. Sensory	0.14	0.09	NA	0.001	0.001	NA
Visual vs. Motor	0.77	0.79	NA	0.29	0.23	NA
Balance vs. Sensory	0.18	0.44	NA	0.02	0.06	NA
Balance vs. Motor	0.67	0.74	NA	0.58	0.61	NA
Sensory vs. Motor	0.47	0.73	NA	0.28	0.57	NA
Visual vs. Balance vs. Sensory vs. Motor	NA	NA	0.43	NA	NA	0.03
